# Evaluating patients with oral leukoplakia in smokers versus non-smokers: A clinico-histopathological study

**DOI:** 10.6026/973206300223137

**Published:** 2026-05-31

**Authors:** Latha M, Lavanya R.M, Megha Modi, Baby Thomas Chacko, Rolly Gupta, Kirti Nishant Vyas, Ramanpal Singh Makkad

**Affiliations:** 1 Department of Oral Pathology, KGF College of Dental Sciences, KGF, Karnataka, India; 2 Department of Oral Pathology, Rajiv Gandhi Dental College, Bengaluru, Karnataka, India; 3 Department of Oral and Maxillofacial Surgery, New York University College of Dentistry, New York, United States; 4 Department of Radiological Science, College of Applied Medical Science, King Saud University, Riyadh, Saudi Arabia; 5 Department of Oral Pathology, Shri Shankaracharya Institute of Dental Sciences, Bhilai, Chhattisgarh, India; 6 Department of Pathology, Dr N D Desai Faculty of Medical Science and Research, Dharmsinh Desai University, Nadiad, Gujarat,India; 7 Department of Oral Medicine and Radiology, New Horizon Dental College and Research Institute, Bilaspur, Chhattisgarh, India

**Keywords:** Oral leukoplakia (OLK), smokers, non-smokers, malignant disorders

## Abstract

Variable clinical behavior is seen in oral leukoplakia, a potentially malignant illness of the mouth that may be caused by etiological factors including cigarette smoking. Therefore, it is of interest to compare the features of oral leukoplakia lesions and epithelial dysplasia in 100 individuals, all of whom were either smokers or non-smokers, using a clinico-histopathological approach. Histopathological analysis was used to ascertain the extent of epithelial dysplasia after a thorough clinical evaluation that included the location, size, shape and duration of the lesion. Smokers were more likely to have leukoplakia on the buccal mucosa, whereas non-smokers tended to have lesions on the gingiva and tongue. Regardless of smoking status, histopathological investigation showed that moderate to severe dysplasia occurred in a considerable number of patients, even though mild dysplasia predominated in both groups. Thus, we show the need of biopsy and routine monitoring of all leukoplakic lesions, even while smoking is a major etiological factor for leukoplakia formation; dysplastic alterations and possible malignant risk may occur in smokers and non-smokers alike.

## Background:

A lesion of the oral mucosa that is mostly white and cannot be diagnosed histopathologically or clinically as any other disease is called oral leukoplakia. There is a known chance that it might progress to oral squamous cell carcinoma, making it the most prevalent potentially malignant oral condition. Worldwide, oral leukoplakia prevalence estimates range from 1% to 5%; groups with a history of cigarette use and other carcinogenic behaviors tend to have higher rates [1,2]. It has long been believed that tobacco use and smoking in particular, is a major contributor to the onset of oral leukoplakia. Tobacco smoke contains carcinogens such as nitrosamines and polycyclic aromatic hydrocarbons, which cause alterations in the oral mucosa known as dysplastic changes, epithelial hyperkeratinization, DNA damage and cellular atypia [3, 4]. Stopping smoking may lead to the regression of some lesions and epidemiological studies have indicated that smokers are far more likely to acquire leukoplakia than non-smokers [5]. Although there is a high correlation between cigarette use and leukoplakia, a large number of instances still occur in people who have never smoked. There is growing interest in leukoplakia in non-smokers due to the possibility that these lesions constitute separate biological entities involving different molecular mechanisms of carcinogenesis. Leukoplakia in non-smokers may have dysplasia and malignant transformation rates that are equivalent to, or even greater than, lesions associated with tobacco use [6, 7]. This finding suggests that other variables, including as predisposition, persistent inflammation, malnutrition, viral infections and immunological dysregulation, could play a role in pathogenesis [8]. Leukoplakia may manifest clinically in a variety of ways, including flat, homogenous plaques, nodules, or verrucous lesions. It has been shown that non-homogeneous lesions are more likely to undergo malignant transformation than homogeneous ones [9]. In addition, the location of the tumor is very important when making a prognosis. Compared to lesions on the buccal mucosa, those on the floor of the mouth, soft palate and tongue typically show a greater dysplastic potential [10]. When it comes to determining how serious leukoplakia is, histopathological assessment is still considered the gold standard. The most accurate indicators of cancerous transformation are the existence and severity of epithelial dysplasia, with transformation rates rising sharply with increasing dysplasia severity [11]. Nevertheless, it is crucial to do regular biopsies on all suspected lesions [12], since clinical presentation is not always enough to predict histological severity. Few researches have directly compared smokers and non-smokers clinico-histopathologically, despite the large number of studies that have looked at leukoplakia in general populations. Helping doctors detect high-risk lesions earlier and modify care options appropriately may be possible by understanding variations in lesion features, anatomical distribution and dysplastic alterations across different groups [13, 143]. Therefore, it is of interest to compare individuals with and without smoking history of oral leukoplakia using a mixed clinical and histological methodology and to enhance early diagnosis of possibly malignant alterations in the oral cavity by comparing lesion appearance and dysplastic severity between habit groups.

## Materials and Methodology:

## Study design and setting:

The Oral Medicine and Oral Pathology Department of a university-affiliated dentistry school carried out this prospective observational clinico-histopathological investigation. Examining the histological features and clinical manifestations of oral leukoplakia in individuals who smoked and those who did not was the primary objective of the research.

## Sample size:

A total of 100 patients clinically diagnosed with oral leukoplakia were included in the study over the study period.

## Study Groups:

Patients were categorized into two groups based on tobacco smoking history:

[1] Group I - Smokers: 50 patients with a history of smoking for at least one year

[2] Group II - Non-smokers: 50 patients with no history of smoking or smokeless tobacco use

## Inclusion criteria:

[1] Patients aged 18 years and above

[2] Presence of clinically diagnosed leukoplakic lesion persisting for more than 2 weeks

[3] Patients willing to undergo biopsy and provide informed consent 

## Exclusion criteria:

[1] Patients with clinically diagnosable white lesions other than leukoplakia (e.g., lichen planus, candidiasis, frictional keratosis)

[2] History of prior treatment for leukoplakia

[3] Patients with systemic diseases affecting oral mucosa

[4] Alcohol dependence without tobacco exposure (to avoid confounding bias)

## Clinical evaluation:

A detailed clinical examination was performed for each patient and the following parameters were recorded:

[1] Demographic details: age, gender

[2] Habit history: duration and frequency of smoking

[3] Lesion characteristics:

1) Site of lesion

2) Size (measured using periodontal probe in mm)

3) Clinical type (homogeneous/non-homogeneous)

4) Surface texture and margins

[4] Duration of lesion as reported by the patient

[5] Lesions were photographed and charted for documentation.

## Histopathological examination:

Under local anesthetic, every patient had an incisional biopsy taken from the lesion's most representative location. The tissue samples underwent standard processing after being fixed in 10% buffered formalin. An oral pathologist who was not aware of the patient's habit status examined the sections stained with hematoxylin and eosin.

Using the World Health Organization's dysplasia grading system, epithelial alterations were assessed as follows:

[1] No dysplasia

[2] Mild dysplasia

[3] Moderate dysplasia

[4] Severe dysplasia / carcinoma in situ

## Outcome measures:

The primary outcomes assessed were:

[1] Distribution of leukoplakia according to site and clinical type

[2] Degree of epithelial dysplasia

[3] Comparison of clinical and histopathological findings between smokers and non-smokers 

## Statistical analysis:

Data were entered into statistical software and analyzed accordingly. Continuous variables were expressed as mean ± standard deviation. Categorical variables were expressed as percentages. Chi-square test was used for comparison of categorical variables. Independent t-test was used for comparison of mean values. A p-value < 0.05 was considered statistically significant.

## Results:

One hundred individuals with oral leukoplakia as determined by clinical examinations were a part of the research. Out of the total number of patients, 50 smoked cigarettes while the other 50 did not. The study population had an average age of 46.2 ± 11.4 years and the smoked group was mostly comprised of males. In comparison to non-smokers, smokers were noticeably older (p = 0.041). While men made up the majority in both groups, smokers' percentage was much greater (78% vs. 56%, p = 0.018). This indicates that middle-aged men are at a higher risk for developing smoking-related leukoplakia (Table 1). Smokers were more likely to have involvement of the buccal mucosa (48% vs. 28%) while non-smokers were more likely to have lesions on the tongue (36% vs. 18%). This suggests that lesions caused by tobacco usually manifest in areas of direct exposure, but lesions in non-smokers may manifest on high-risk mucosa like the tongue (Table 2). In both groups, homogeneous leukoplakia was the most prevalent symptom. Non-smokers had a little higher incidence of non-homogeneous lesions (40% vs. 32%), but the difference was not statistically significant. This tendency has important clinical implications since lesions that are not homogenous are more likely to become cancer (Table 3). The most common histological finding in both groups was mild dysplasia. It is possible that leukoplakia in non-smokers is not inherently less aggressive, as moderate-to-severe dysplasia was somewhat more prevalent among non-smokers (40%) compared to smokers (30%). Having said that, there was no statistical significance in this distinction (Table 4).

## Discussion:

Despite comparable dysplasia rates, this clinico-histopathological investigation contrasting oral leukoplakia in smokers and non-smokers reveals many noteworthy disparities in lesion morphology, demographic distribution and possible malignant aspects. Tobacco carcinogens are directly exposed to the oral cavity and tend to harm regions that come into touch with smoke. As a result, smokers had higher lesions on the buccal mucosa (48% vs. 28%) compared to non-smokers. There was a significant difference between smokers and non-smokers in terms of the proportion of lesions in high-risk areas like the tongue (36% vs. 29% and 23%, respectively). This finding has important therapeutic implications because lesions in these places are more likely to undergo malignant transformation (MT) for many reasons. These results are in line with previous research from a large comparative cohort that found that non-smokers were more likely to have involvement of the tongue and gums than smokers (p = 0.016) [15]. Despite smoking's well-documented association with an increased risk of leukoplakia, this study's histopathological examination revealed no statistically significant difference in the prevalence of epithelial dysplasia between the smoking and non-smoking groups (43.5% vs. 39.1%, respectively). Although the occurrence of dysplasia determines the likelihood for malignant transformation, this finding is consistent with previous clinical findings in which smoking status did not correlate substantially with the severity of dysplasia [16]. It is worth noting that in multiple cohorts, the malignant transformation rate was nearly twice as high in non-smokers as in smokers. This finding raises the possibility that non-smokers' progression is influenced by factors other than tobacco use, such as their genetic predisposition, immune status and lesion behavior. Although the exact processes are still unknown, recent clinical treatment studies have shown that non-smoker leukoplakia, particularly those that are female-predominant or located on high-risk sites (such the tongue), may be more likely to undergo metamorphosis [11, 12]. Cigarette smokers have a prevalence of oral leukoplakia that is 3.2-6 times greater than non-smokers, according to previous epidemiological investigations, highlighting tobacco as one of the most prevalent risk factors for leukoplakia development. Nonetheless, dysplasia risk, anatomical location, clinical manifestation (homogeneous vs. non-homogeneous) and prevalence differ across groups. These details highlight the need of assessing leukoplakia using clinical and histological criteria, irrespective of smoking status [10, 13]. Lesion size, non-homogeneous morphology, advanced age and female gender are other characteristics that have been associated with an elevated risk of malignancy in various meta-analyses. The presence of nodularity or a mixture of red and white patches in non-homogenous lesions remains a powerful indicator of potential malignant development [16]. While histological severity and malignant potential are evident in both smokers and non-smokers, this research adds to the current data that smoking affects lesion location and demographic presentation. Since dysplastic alterations and transformation may happen regardless of smoking status, our results highlight the clinical importance of biopsy and diligent follow-up for all leukoplakia lesions, regardless of habit history.

## Conclusion:

Leukoplakia on the buccal mucosa was more common in smokers than non-smokers in this sample of 100 individuals, although lesions on high-risk locations including the tongue were more common in non-smokers. The necessity for careful observation and histological evaluation of all oral leukoplakias irrespective of smoking status is underscored by the fact that, although dysplasia frequency was comparable across groups, non-smokers exhibited a tendency toward greater malignant development.

## Figures and Tables

**Table 1:** Distribution of patients according to age and gender

**Variable**	**Smokers (n=50)**	**Non-smokers (n=50)**	**Total**	**p-value**
Mean age (years)	48.6± 10.2	43.8± 12.1	-	0.041*
Males	39 (78%)	28 (56%)	67 (67%)	0.018*
Females	11 (22%)	22 (44%)	33 (33%)	

**Table 2:** Site distribution of leukoplakia

**Site of lesion**	**Smokers (n=50)**	**Non-smokers (n=50)**	**Total (%)**	**p-value**
Buccal mucosa	24 (48%)	14 (28%)	38%	0.029*
Tongue	9 (18%)	18 (36%)	27%	0.036*
Gingiva	7 (14%)	10 (20%)	17%	0.41
Floor of mouth	6 (12%)	5 (10%)	11%	0.75
Other sites	4 (8%)	3 (6%)	7%	0.69

**Table 3:** Clinical type of leukoplakia

**Clinical Type**	**Smokers (n=50)**	**Non-smokers (n=50)**	**Total (%)**	**p-value**
Homogeneous	34 (68%)	30 (60%)	64%	0.39
Non-homogeneous	16 (32%)	20 (40%)	36%	0.37

**Table 4:** Histopathological grading of epithelial dysplasia

**Dysplasia Grade**	**Smokers (n=50)**	**Non-smokers(n=50)**	**Total (%)**	**p-value**
No dysplasia	14 (28%)	12 (24%)	26%	0.65
Mild dysplasia	21 (42%)	18 (36%)	39%	0.54
Moderate dysplasia	11 (22%)	13 (26%)	24%	0.63
Severe dysplasia	4 (8%)	7 (14%)	11%	0.34
